# The Role of Endoscopic Ultrasound and Ancillary Techniques in the Diagnosis of Autoimmune Pancreatitis: A Comprehensive Review

**DOI:** 10.3390/diagnostics14121233

**Published:** 2024-06-12

**Authors:** Flavio Metelli, Guido Manfredi, Nico Pagano, Elisabetta Buscarini, Stefano Francesco Crinò, Elia Armellini

**Affiliations:** 1 Gastroenterology and Endoscopy Department, ASST Maggiore Hospital Crema, 26013 Crema, Italy; flavio.metelli@asst-crema.it (F.M.); guido.manfredi@asst-crema.it (G.M.); elisabetta.buscarini@asst-crema.it (E.B.); 2 Gastroenterology Unit, Department of Oncological and Specialty Medicine, University Hospital Maggiore della Carità, 28100 Novara, Italy; nico.pagano@maggioreossp.novara.it; 3 Diagnostic and Interventional Endoscopy of Pancreas, Pancreas Institute, University of Verona, 37134 Verona, Italy; stefanofrancesco.crino@ospedaleuniverona.it; 4 Gastroenterology and Endoscopy Unit, ASST-Bergamoest, 24068 Seriate, Italy

**Keywords:** autoimmune pancreatitis, endoscopic ultrasound, contrast-enhanced endoscopic ultrasound, time–intensity curves, elastography, fine-needle aspiration, fine-needle biopsy, chronic pancreatitis, pancreatic cancer

## Abstract

Autoimmune pancreatitis (AIP) is a unique form of chronic pancreatitis with a multifactorial pathogenesis. Historically, it has been classified as type 1 and type 2, according to its clinical and histological features. The diagnosis of AIP is challenging and relies on a combination of clinical, histopathologic, serologic, and imaging characteristics. In the available guidelines, the imaging hallmarks of AIP are based on cross-sectional imaging and cholangiopancreatography retrograde endoscopic findings. Endoscopic ultrasound (EUS) is generally used for pancreatic tissue acquisition to rule out pancreatic cancer and diagnose AIP with limited accuracy. Several papers reported the reliability of EUS for providing informative morphologic features of AIP. Nowadays, the improvement in the resolution of EUS conventional images and the development of new ancillary technologies have further increased the diagnostic yield of EUS: contrast-enhanced EUS and EUS elastography are non-invasive and real-time techniques that strongly support the diagnosis and management of pancreatic diseases. In this review article, we will present the role of conventional EUS and ancillary diagnostic techniques in the diagnosis of AIP to support clinicians and endosonographers in managing this condition.

## 1. Introduction

Autoimmune pancreatitis (AIP) is a unique form of chronic pancreatitis (CP) with a multifactorial pathogenesis involving the interplay of immunological, genetic, and environmental factors [1]. It accounts for 5–6% of all cases of CP [2]. The concept of AIP was first proposed in 1995 by Japanese investigators who described a case of CP characterized by autoimmune manifestations on laboratory, histopathological, and clinical findings [3]. Since then, the increasing awareness of the condition has led to several reports worldwide and the recognition of AIP as a distinct clinical entity [4]. AIP has been classified into type 1 (AIP1) and type 2 (AIP2) based on histological and clinical characteristics. A new type of AIP, termed type 3 AIP, induced by immunotherapy, has recently emerged but is still gaining acceptance in the medical literature [5].

AIP1 is referred to as lymphoplasmacytic sclerosing pancreatitis and is recognized to be the pancreatic manifestation of immunoglobulin (Ig)G4-related systemic disease, characterized by increased levels of serum IgG4 and multifocal IgG4-rich lymphoplasmacytic infiltrate associated with intense sclerosis [6,7]. The most frequently involved organs are the pancreatic–hepatobiliary tract (45%), the major salivary glands (37%), the lacrimal gland (26%), the retroperitoneum (15%), the kidneys (15%), the lungs (14%), and the aorta (10%) [8]. Additionally, the focal infiltration of IgG4-positive plasma cells has been detected endoscopically in the stomach, duodenum, and colon [9]. This type of AIP is the most common in Asia, and it presents with obstructive jaundice in three-quarters of cases, especially in elderly males [10]. If other classic diagnostic criteria are present, the diagnosis of AIP1 can be made without pancreatic biopsies [11,12].

AIP2 is characterized by a distinct histology termed idiopathic duct-centric pancreatitis with granulocytic epithelial lesions, commonly observed in Europe and the United States. In comparison with AIP1, AIP2 presents more often with abdominal pain and acute pancreatitis, occurs at a younger age (4th–5th decade), and shows no gender predilection [11]. Further, this type of AIP is pancreas-specific and has little or no association with IgG4 activity, requiring an adequate histological specimen to make a definitive diagnosis [13].

The standard therapy for AIP is based on steroids, with a very high response rate (>95% of cases). After inducing the remission of the disease, relapses are seen in around 30% of AIP1 patients and only in 9% of AIP2 patients [14].

The diagnosis of AIP is challenging and relies on a comprehensive work-up that includes different data. Several groups from different countries proposed diagnostic criteria over the years [5,15,16,17,18]. In 2012, the International Association of Pancreatology proposed the International Consensus Diagnostic Criteria (ICDC) [19], which are the most widely used. The ICDC combine the five cardinal features of AIP: (1) the imaging of the pancreatic parenchyma and pancreatic duct, (2) serology (serum IgG4), (3) other organ involvement, (4) the histology and IgG4 immunostaining of the pancreas, and (5) the response to corticosteroids. Different from the other criteria, the ICDC allows for the diagnosis of both subtypes of AIP independently and can be applied worldwide in clinical and research practice. In addition, the ICDC includes the criteria for AIP not otherwise specified for those cases not clearly defined as either AIP1 or AIP2.

Guidelines support the preoperative differentiation of AIP with pancreaticobiliary malignancies that is crucial to avoid the consequences of progressive disease [20,21,22] and unnecessary surgery [23,24,25]. The focal form of AIP, accounting for 28–41% of AIP cases, poses a clinical challenge when presenting with pancreatic mass, enlargement, or prominence, particularly in the pancreatic head, potentially leading to biliary obstruction and resembling malignancies such as pancreatic cancer (PC) or lymphoma [26,27,28]. Bile duct wall thickening and strictures resulting from IgG4 sclerosing cholangitis, the main extrapancreatic manifestation in patients with AIP, can mimic both primary sclerosing cholangitis and cholangiocarcinoma (CCA) [29,30,31].

In the available guidelines, the imaging features of AIP are depicted using cross-sectional imaging systems [6,19] and endoscopic retrograde cholangiography [15,16,17,18], while endoscopic ultrasound (EUS) is mainly performed to obtain tissue samples to rule out PC [32,33,34].

Nevertheless, several papers have reported the reliability of EUS in providing the morphological features of pancreatic disease in patients with AIP [35,36,37,38]. However, there is still a lack of standardization.

Recent Japanese consensus criteria highlighted the relevance of some EUS imaging features to differentiate between AIP, PC, and CP [39], but efforts must continue in this direction. Nowadays, the progress in terms of the resolution of conventional images and the development of ancillary techniques, such as elastography and contrast enhancement, has further increased the diagnostic yield of EUS. Data from artificial intelligence and EUS-based convolutional neural network models show promise in the differentiation of AIP and pancreatic masses, but their implementation in clinical practice is still in an early stage [40,41].

In this review article, we will present the role of conventional EUS and ancillary diagnostic techniques in the diagnosis of AIP with the purpose of providing a practical tool for clinicians and endosonographers to manage this condition.

## 2. Conventional EUS

### 2.1. Diagnosis

The imaging appearances of AIP can vary markedly, depending on multiple factors including the degree of fibrosis and inflammatory infiltrate. Diffusely or locally enlarged pancreas with the distortion and/or loss of the lobular architecture (termed “sausage-shaped pancreas”), a capsule-like rim, and a distinctive delayed enhancement pattern are the characteristic findings of cross-sectional imaging in AIP [39,42]. In conventional EUS imaging, the most characteristic finding in AIP is diffuse pancreatic enlargement with a hypoechoic, patchy, heterogeneous echotexture [36,43,44,45]. Less frequently, EUS may reveal focal hypoechoic enlargement and/or solitary or diffuse hypoechoic areas as a result of localized pancreatic involvement [46]. Many EUS features useful for the diagnosis of AIP have been reported with a certain trend in the literature [35,37,43,44,45,47,48].

**Figure 1:**
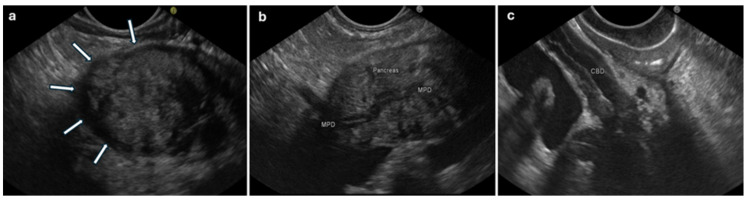
Endoscopic ultrasonography in autoimmune pancreatitis. (**a**) Hypoechoic rim around pancreatic parenchyma (arrows). (**b**) Visible unobstructed main pancreatic duct (MPD) with thickened wall: “duct-penetrating sign”. (**c**) Thickened wall of common bile duct (CBD) with 3-layer-type appearance.

In a recent large retrospective study [37], Sheng-Yu Zang et al. explored the EUS features of 285 patients of newly diagnosed type 1 AIP and compared the typical AIP and CP features between the diffuse (d-AIP)- and focal (f-AIP)-type AIP. The Rosemont criteria [49] were employed for CP feature definition. There were 214 cases of d-AIP (defined as “enlargement involving > 1/2 of pancreas”) and 71 cases of f-AIP (defined as “enlargement involving ≤ 1/2 of pancreas”); among focal cases, more lesions were in the pancreatic head (50 cases, 70.4%). First, they reported the more common typical EUS features in AIP which were consistent with previous studies: diffuse hypoechoic area (74.7%), bile duct wall thickening (68.4%) or stenosis (57.9%), peripancreatic lymphadenopathy (31.2%), peripancreatic hypoechoic margin (28.4%), and lobular outer margin (14%). The more common CP changes reported were hyperechoic foci (95.1%), hyperechoic strands (61.1%), a hyperechoic main pancreatic duct (MPD) margin (41.8%), lobularity (25.9%), MPD dilation (16.8%), and diffuse MPD stenosis/irregularity (10.2%). For the typical AIP features, there were significantly more patients in the diffuse group with diffuse hypoechoic areas (92.1% vs. 22.5%, *p <* 0.001), bile duct wall thickening (73.4% vs. 52.1%, *p =* 0.001), and peripancreatic hypoechoic margin (35.5% vs. 7.0%, *p* < 0.001) (Figure 1a). For the CP features, there were significantly more patients in the focal group with MPD dilation (14.0% vs. 25.3%, *p* = 0.03).

Tao Guo et al. [48] retrospectively examined the EUS characteristics between 90 patients with f-AIP, 127 with d-AIP, and 197 patients with PC. The authors divided the pancreas into three parts to define d-AIP and f-AIP: head, body, and tail; cases with more than one part of the pancreas enlarged were defined as “diffuse enlargement” and grouped as d-AIP; cases with less than one part of the pancreas enlarged were defined as “focal enlargement” and grouped as f-AIP. Focal hypoechogenicity and MPD dilation were more frequent in f-AIP patients, whereas diffuse hypoechogenicity was more common in d-AIP patients. No significant difference was found in the frequency of other EUS features between the two groups instead. The authors noted that among 90 patients with f-AIP, 70 patients (77.8%) showed pancreatic diffuse hypoechogenicity. However, the form of the body and tail was not enlarged compared with a prominent enlargement of the head, and only 19 patients (21.1%) showed focal hypoechogenicity confined to the head.

### 2.2. Differences with PC on B-Mode

In f-AIP, a solitary hypoechoic mass lesion with irregular margins, usually located in the pancreatic head, was observed [35,37]. In certain cases, the pancreas is devoid of hyperechoic strands and lobularity, and it influences the surrounding blood vessels [47]. AIP can also cause peripancreatic lymphadenopathy and pseudovascular invasion. Thus, it can be very difficult to differentiate AIP from PC on the basis of imaging only, and this still represents a subject of scientific investigation.

In the aforementioned study, Tao Guo et al. found that diffuse hypoechogenicity, hyperechoic foci/strands, lobularity, peripancreatic hypoechoic margin, and bile duct wall thickening were significantly more frequent in f-AIP compared to PC. Conversely, focal hypoechogenicity, MPD dilation, and vascular invasion were more characteristic of PC.

In the study by Tacelli M et al. [50], the “duct-penetrating sign”, defined as a visible unobstructed PD that penetrates the mass, was another important feature observed more frequently in f-AIP than in PC patients (66.7% vs. 11.7%, *p* < 0.001) (Figure 1b). These results agree with those found in a previous study by Hoki et al. [35], in which 25 AIP patients and 30 patients with PC were assessed by EUS and other imaging modalities. Interestingly, despite the small sample size and simple design that were the limitations of this study, in the comparison among EUS, transabdominal ultrasound, and computed tomography (CT), EUS was far superior for revealing lymphadenopathy (72%, 4%, and 8%, respectively; *p* < 0.001) and the duct-penetrating sign (24%, 4%, and 0%, respectively; *p* < 0.01).

### 2.3. Staging

Since AIP is a chronic disease, EUS may also be useful to determine its stage and activity. Kubota K et al. [51] reported that EUS imaging features differ between early-stage AIP and advanced-stage AIP, based on the Cambridge classification [52] for CP. Lobularity and a hyperechoic pancreatic duct margin were detected at a significantly higher frequency in early-stage AIP, demonstrating histologically interlobular and periductal fibrosis in pancreatic parenchyma with acinar cells preserved. Other EUS findings, such as hyperechoic foci and hyperechoic strands, were recognized in both stages. This study suggests a potential role of EUS in detecting the characteristics of early-stage AIP, which is characterized by a prompt response to corticosteroid therapy or may benefit from a wait-and-watch strategy given the possibility of spontaneous remission [14,53].

In the advanced stage, AIP can lead to pancreatic stones and cystic space formation, glandular atrophy, and/or the irregular dilatation of the MPD, similar to those described in other forms of CP [51,54]. In their review article, Maruyama M et al. [55] stated that approximately 40% of AIP patients experience pancreatic stone formation over a long-term course, and nearly 20% progress to confirmed CP according to the JCDC criteria [16]. They also observed that pancreatic calcification in AIP is closely associated with disease recurrence. However, in the study by Hoki et al. [35], the Sahai criteria [56] for CP were found to be not sensitive to the diagnosis of AIP since the 25 patients included were classified as normal or displaying mild disease according to the scoring system. Similarly, only a small proportion of newly diagnosed AIP patients were classified as “suggestive of CP” and even rarer “consistent with CP” via the Rosemont criteria [49] in the large retrospective study mentioned above [37]. The most likely explanation is that CP change is relatively limited for most newly diagnosed AIP cases that were probably in the early stage of the disease. Moreover, these data support the assumption that the process of fibrosis development in AIP differs from what is observed in other forms of CP [47].

### 2.4. Therapeutic Monitoring

Steroid therapy is very effective for AIP, and therefore, the imaging findings may differ before and after treatment [47,51]. In the study reported by Okabe et al. [47], EUS was performed before and within 2 weeks of beginning steroid therapy in 14 AIP patients. They found that hyperechoic strands, lobularity, and lobular outer margins rapidly improved or were eliminated in more than 50% of patients, although hyperechoic foci persisted. Lobularity improved in more than 70% of patients. The improvement in the findings after steroid therapy serves as evidence for the benefit of such therapy and may also support differential diagnosis from PC.

### 2.5. MPD, Biliary, and Peripancreatic Findings

The MPD may also be narrowed with wall thickening in areas of pancreatic involvement [43,57,58]. When present, the upstream dilation of the MPD is typically milder than what is observed in patients with PC [38]. However, MPD changes in AIP are best demonstrated on MR and/or MRCP. According to guidelines, typical characteristics are the long narrowing of the MPD (>one-third of the entire length) and lack of significant upstream dilatation (<5 mm) [19,39]. In some cases, multiple stenotic (“skip”) lesions are observed. The irregular narrowing of the MPD characterized by alternating collapsed and normal segments in association with MPD wall thickening at EUS showed an overall accuracy of 98.3% for AIP [59]. If the narrowing is localized and/or the upstream dilatation of the MPD is present, differential diagnosis with PC should be considered. In such cases, findings such as side branches arising from the narrowed area or skipped narrowed lesions are useful for differentiating the disease from PC [39,60,61].

As the biliary tree is the most common organ involved other than the pancreas in AIP (up to 80% of patients show stenosis of the bile duct, most commonly in the distal region), it is important to evaluate the extrahepatic duct during EUS. Bile duct and gallbladder wall thickening, intrapancreatic bile duct stricture, and extrahepatic bile duct dilation are the most common EUS findings [30,31,42]. In a study of 37 patients with AIP, the EUS findings of extrahepatic bile duct and gallbladder wall thickening were observed in 38% [61]. Two types of bile duct thickening were identified: the 3-layer type (64.3%) with a high–low–high echo appearance and a parenchymal echo type (35.7%) with a thickened wall that occupied the entire bile lumen with appearances of a parenchymal echo in the bile duct. Intrahepatic bile duct wall thickening and gallbladder wall thickening were detected in seven and nine patients, respectively. A similar appearance to the 3-layer type with a regular homogenous thickening with a hyper–hypo–hyperechoic series of layers of the ductal wall (the so-called “sandwich pattern”) was seen on EUS in different series [57,62] (Figure 1c).

Intraductal ultrasonography (IDUS) is, therefore, useful in the evaluation of the bile duct wall and can provide further information for the differentiation of IgG4 sclerosing cholangitis (SC) from CCA and primary sclerosing cholangitis [63,64,65]. The characteristic IDUS findings in AIP are circular-symmetric wall thickness with a smooth configuration of the outermost layer and a smooth luminal surface [64,66]. In contrast, the sonographic features of CCA on IDUS include eccentric wall thickening with an irregular luminal surface, the disruption of the layer structure of the bile duct wall, and a hypoechoic mass with irregular margins [66]. In a retrospective study, Naitoh et al. [67] found that the thickening of the bile duct wall (exceeding 0.8 mm) in the nonstenotic region on a cholangiogram is a specific IDUS finding for differentiating AIP from CCA. This IDUS feature had a sensitivity, specificity, and accuracy of 95%, 100%, and 93.5%, respectively, for differentiating IgG4-SC from CCA. However, Kuwatani M. et al. [68] stated that IDUS findings alone are insufficient for differentiation between IgG4-SC and CCA since the IDUS findings of IgG4-SC were found to be various, and those of both IgG4-SC and CCA overlap each other to some extent. Finally, the diagnosis of bile duct stenosis may be difficult, requiring a comprehensive combination of clinical, radiological, and histopathological data [69,70].

Other EUS features of AIP include peripancreatic lymphadenopathy and vascular invasion. EUS can reveal single or multiple enlarged lymph nodes in patients with AIP, reflecting the underlying inflammatory process, which can involve organs other than the pancreas [35,71]. The frequency of lymphadenopathy in AIP patients is reported with high variability between studies on EUS, ranging from 31.2% to 72% [35,37,48]. Interestingly, a trend toward a higher prevalence of lymphadenopathy in AIP compared to PC was observed [48]. Hoki et al. [35] reported a significant superiority of EUS in the detection of lymphadenopathy over CT (72% vs. 8%) in patients with AIP. However, the conventional EUS criteria of metastatic nodes, including size >1 cm, hypoechoic appearance, round shape, and smooth borders [72], have proven inaccurate in pancreaticobiliary cancers [73,74]. Considering this and the lack of specific EUS features of lymph nodes in AIP patients, differential diagnosis with biliopancreatic malignancy may be arduous.

Regarding peripancreatic vascular involvement, it is a common finding detected in 44–67% of AIP patients in series using CT [75,76,77]. In two studies [75,76], peripancreatic vascular involvement was predominantly recognized in d-AIP compared to f-AIP (*p* = 0.033 and *p* = 0.06), while in one study [77], this was significantly associated with the location of the pancreatitis lesion including the pancreatic tail (*p* = 0.01). Of the specific vascular lesions observed, stenosis and/or the occlusion of the splenic vein and/or superior mesenteric–portal vein, and peri-gastric collateral circulation formation are the most common. Stenosis of the splenic or superior mesenteric artery is also described in a few cases. EUS has a sensitivity and specificity of 85% and 91%, respectively, in diagnosing vascular invasion by PC [78], and peripancreatic major vein invasion is more accessible by EUS than by CT [79]. Of note, in the two largest retrospective series of patients with AIP, EUS detected peripancreatic vessel involvement in only 19.3% [48] and 7.4% [37] of cases, respectively. No difference between diffuse- and focal-type AIP was found [37]. Moreover, vascular lesions were not specified. Only one study [48] reports the definition of vascular involvement in terms of the loss of interface between the pancreas and vessels of the portal system. Some reasons can explain this different frequency in vascular involvement detection between cross-sectional imaging and EUS. First, endosonographers may focus more on biliopancreatic changes during the EUS evaluation of AIP since they are faced with benign disease, especially for the diffuse form. The swelling of the pancreatic parenchyma in the diffuse form can make the assessment of peripancreatic vessels by EUS more difficult. Additionally, cross-sectional imaging provides a standardized method for patient evaluation, which is not operator-dependent, and images are typically stored and can be reinterpreted later by different observers for research purposes.

In summary, conventional EUS has been demonstrated to be a useful tool for evaluating the morphologic features of AIP with implications for diagnosis, staging, therapeutic monitoring, and differentiation with other pancreatic diseases including PC. The main EUS findings in AIP are summarized in Table 1.

We observed that most of the studies on the topic are retrospective and do not distinguish between type 1 and type 2 AIP nor between the diffuse and focal types when considering the characteristics of the disease on conventional EUS. The studies by Sheng-Yu Zang et al. [37] and Tao Guo et al. [48] tried to explore and compare the EUS characteristics of diffuse and focal forms of AIP. Another emerging issue is the lack of a standardized definition for focal and diffuse forms of AIP on EUS.

However, the main goal for clinicians is to distinguish between AIP, specifically the focal form, and pancreatic malignancies as PC. As reported, in some cases, the clinical and radiological findings of AIP and PC may overlap making it arduous to reach a diagnosis. For this purpose, conventional EUS can display important information during the initial evaluation of this subgroup of patients but is often not enough despite high sensitivity and specificity over cross-sectional imaging in the detection of pancreatic malignancies [80]. Nevertheless, in challenging clinical scenarios, EUS ancillary techniques provide crucial support for diagnosis including a guide to pancreatic biopsies, which are often required.

## 3. Contrast-Enhanced EUS

The use of contrast agents for ultrasonography provided the opportunity for hemodynamic analysis during conventional EUS [81], and the usefulness of CE-EUS in the diagnosis of pancreatic diseases has been widely reported [81,82,83,84,85,86,87]. Today, the technique of CE-EUS relies on a dedicated contrast harmonic echo (CH-EUS) that can detect signals from microbubbles delivered by second-generation contrast agents, even in vessels with very slow flow [88]. CH-EUS provides the clear real-time blood vessel imaging of a target tissue without the artifacts encountered with Doppler modes. Moreover, it also allows for the delineation of the time–intensity curves (TICs) and graphing of the changes in brightness over time through contrast [89].

A meta-analysis considering 12 studies on CE-EUS in the diagnosis of PC indicated a pooled sensitivity and specificity for PC of 94% and 89%, respectively [90]. Since the differential diagnosis of AIP and PC is challenging, CH-EUS may play an important role in this purpose. Previous studies have shown that the isovascular homogeneous pattern on CH-EUS is a characteristic of pancreatic inflammatory mass lesions, including AIP [91,92,93]. This pattern corresponds with homogeneous pancreatic cells with abundant vessels and no necrotic or fibrous tissues in contrast with heterogeneous tumor cells with necrotic, fibrous tissue and few vessels present in PC [92]. Consequently, CE-EUS features such as isoenhancement or hypoenhancement, arterial irregularity, and absent venous vasculature within a mass-forming PC [81].

**Figure 2:**
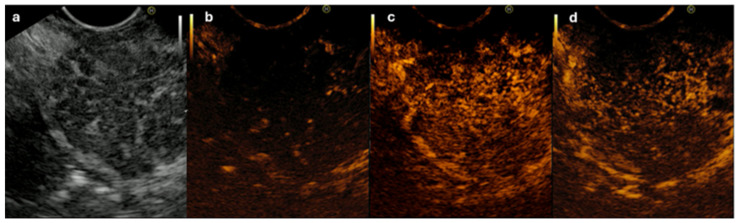
Contrast-enhanced harmonic EUS of focal-type autoimmune pancreatitis. (**a**) Hypoechoic focal enlargement of pancreatic head (B mode). (**b**) Second harmonic imaging before contrast. (**c**) Homogeneous hyperenhancement in arterial phase (15 s). (**d**) Homogeneous isoenhancement in late phase (35 s).

Dong et al. [38] retrospectively reviewed 60 histologically confirmed cases of AIP, comparing them to 16 cases of PC. After the injection of Sonovue^TM^, most AIP lesions displayed focal or diffuse isoenhancement (86.6%) in the arterial phase, while most of the PC lesions (93.7%) were hypoenhancing (*p* < 0.01). During the late phase, most AIP lesions were hyperenhanced (65%) or isoenhanced (35%), while most PC lesions were hypoenhancing (93.7%). Cho et al. [94] evaluated the usefulness of CH-EUS for the differential diagnosis of f-AIP and PC (53 PC patients and 27 f-AIP patients). Hyper- to isoenhancement in the arterial phase (89% for f-AIP vs. 13% for PC, *p* < 0.05), homogeneous contrast agent distribution (81% for f-AIP vs. 17% for PC, *p* < 0.05), and absent irregular internal vessels (85% for f-AIP vs. 30% for PC, *p* < 0.05) were observed more frequently in the f-AIP group (Figure 2). The combination of hyper- to isoenhancement and absent irregular internal vessels raised the specificity to 94% in differentiating f-AIP from PC.

TIC analysis is an emerging standardized quantification tool to define the perfusion characteristics of target tissues [95]. The rapid postprocessing of recordings allows trained physicians to objectively analyze otherwise qualitative data provided by contrast enhancement techniques [96,97]. During the intravenous administration of contrast, a continuous cine-clip of a target organ’s enhancement is obtained and saved for later postprocessing. The optimal length of time to observe depends on the target organ and the operator’s choice. A TIC curve is produced for each region of interest (ROI), from which quantitative perfusion parameters can be extracted such as peak intensity, time to peak, wash-in and wash-out rate, area under the curve, and others [98,99]. For this purpose, several commercial solutions exist, either embedded in ultrasound scanners or as stand-alone computer programs [100]. Several studies demonstrated the usefulness of TIC analysis for the evaluation of the microvasculature of pancreatic diseases during CE-EUS, supporting the differential diagnosis of focal pancreatic masses [87,97,100,101]. Recently, TIC analysis has been reported to significantly improve diagnostic yield relative to unenhanced EUS (OR, 10; 1.4–434.0) for solid pancreatic lesions [99].

Matsubara et al. [91] performed CH-EUS using a TIC-based quantitative analysis for the differential diagnosis of pancreatic diseases in 48 patients with PC, 14 patients with AIP, 13 patients with mass-forming pancreatitis, and 16 patients with pancreatic neuroendocrine neoplasms. The authors found significant differences in the echo intensity reduction rates from the peak at 1 min between PC and other diseases, and the sensitivity, specificity, and accuracy of EUS characteristics in combination with TICs were 95.8%, 92.6%, and 94.7%, respectively. Interestingly, no significant differences were noted in the rate of contrast imaging-induced elevation in the echo intensity or velocity of elevation from the rise to peak contrast among the four groups.

Imazu et al. [93] evaluated eight patients with AIP and twenty-two patients with PC who underwent CH-EUS and were assessed by TICs. The peak intensity and maximum intensity gain of AIP lesions were significantly higher as compared with PC lesions (21.4 dB vs. 9.6 dB, 17.5 vs. 6.6). ROI characteristic analysis yielded an optimal maximum intensity gain cut-off value of 12.5 with high sensitivity and specificity. However, TIC analysis requires technically demanding procedures, specialized software, and a careful choice of the ROI to avoid inaccurate estimations.

Given these limitations, Ishikawa T et al. [102] proposed a simplified method by evaluating multiple phases (20, 40, and 60 s) of CE-EUS in the diagnosis of pancreatic solid lesions. They evaluated retrospectively 210 patients with pancreatic solid lesions, including 142 patients with PC and 24 patients with mass-forming pancreatitis (including 20 patients with f-AIP), who underwent CE-EUS and had information on final diagnoses. In the PC group, most of the lesions showed a hypovascular pattern at 20 or 40 s after the injection of contrast medium following early enhancement, consistent with the previous TIC evaluation results. In the mass-forming pancreatitis group (20/24 lesions were f-AIP), the lesions showed various enhancement patterns, and no specific pattern was detected for the diagnosis of AIP using this multiphase evaluation method. However, there was a tendency to show an isovascular pattern at each phase, and considering those who showed at either 20 or 40 s, the sensitivity, specificity, and accuracy were 70.8%, 67.7%, and 68.1%, respectively.

In summary, specific contrast-enhanced vascular patterns are associated with AIP (hyper- or isovascular homogeneous, absent irregular internal vessels) and PC (hypovascular, irregular, and/or absent internal vessels). Again, it is necessary to consider that some AIP cases may have a contrast enhancement pattern similar to that of PC, making the diagnosis challenging. TIC analysis is a promising tool that may further improve the differentiation of focal pancreas lesions including AIP, but a prospective validation of quantitative parameters is needed.

## 4. EUS–Elastography

EUS-EG is a real-time method that measures elasticity by the registration of differences in the distortion of the EUS image after applying pressure by the EUS probe. It can be evaluated qualitatively (i.e., based on color map distribution) or quantitatively (i.e., by quantifying the strain ratio (SR) or strain histogram). Several studies have demonstrated the usefulness and diagnostic yield of EUS-EG, mostly in the context of biliopancreatic disease [103,104,105,106]. EUS recently included shear wave elastography (SWE) which provides absolute values to calculate tissue elasticity objectively, overcoming the main drawback of strain elastography due to the subjective selection of images for color map analysis [107]. Despite being a promising technique, EUS-EG evidence supporting its use in AIP is limited.

In 2009, Dietrich et al. [108] assessed five patients with f-AIP by qualitative EUS-EG describing a recognizable homogenous stiffness of the whole pancreatic parenchyma, not just in the mass but in all of them. This finding was not found in 17 patients with ductal adenocarcinoma or 10 healthy subjects

As mentioned, concerning conventional EUS imaging, the EUS findings of AIP may change after steroid therapy. Ishikawa et al. [109] investigated the usefulness of EUS-EG combined with SR in the estimation of short-term treatment effects in patients with AIP. The EUS-EG images of 10 patients with AIP before and after steroid treatment for 2 weeks were assessed. All 10 patients showed a decrease in the SR at 2 weeks, with statistical significance (8.04 ± 2.29 to 3.44 ± 1.97; *p* < 0.0001), although the initial SR values varied across a wide range. Most of them also showed a significant decrease in the mean size of the pancreas (20.08 ± 2.46 mm to 15.9 ± 2.49 mm; *p* = 0.001), but two cases remained the same size at 2 weeks, suggesting that the elasticity of the pancreas may change before radiological improvement in pancreatic swelling.

EUS-SWE is based on a Doppler-like ultrasound technique to monitor the propagation of shear waves and measure their velocity. Theoretically, greater tissue elasticity corresponds to faster shear wave propagation. The shear wave velocity (Vs), as an elastic module, is measured in a target lesion [110].

The study by Ohno et al. [111] investigates the feasibility and clinical usefulness of EUS-SWE in the diagnosis and treatment of AIP. Eight patients with AIP showed a significantly higher median vs. the sixteen healthy controls (2.57 m/s vs. 1.89 m/s) (*p* = 0.0185). In addition, among the six patients with AIP who underwent steroid therapy, the mean significantly decreased from 3.32 to 2.46 m/s (*n* = 6) (*p* = 0.0234), suggesting a role of EUS-SWE for assessing the effect of steroid therapy.

Ohno et al. [112] also evaluated the feasibility of EUS-SWE for the measurement of the elasticity of solid pancreatic lesions, including 43 cases of PC and 9 cases of mass-forming pancreatitis. However, the median vs. values were 2.19 for PC and 2.56 for mass-forming pancreatitis, with no significant difference. Table 2 summarized the main characteristics EUS findings of AIP in comparison with PC.

Finally, EUS-SWE is a promising imaging technique with advantages in terms of the consistency of results, the objectivity of the method, and diagnostic accuracy compared to the features of strain EG. The evidence suggests its potential role in different clinical scenarios including AIP, but further studies are awaited.

## 5. EUS-Guided Tissue Acquisition

Pancreatic biopsy sampling ideally represents the gold standard for diagnosing AIP, distinguishing it from PC and delineating between type 1 and type 2 AIP. According to the ICDC [19], only tissue samples obtained through core biopsy and surgical resection are suitable for the histological diagnosis of AIP [114].

The Japanese consensus guidelines for AIP proposed in 2020 [39] suggested employing both EUS–fine-needle aspiration (FNA) and EUS-guided fine-needle biopsy (EUS-FNB) in cases where a diagnosis cannot be made without pathology specimen collection or when malignancy is suspected or cannot be ruled out. The ICDC [19] distinguish level 1 or 2 histological criteria according to the presence of typical histological features for both types of AIP (Table 3).

A definitive diagnosis of AIP based on histology is achieved only with level 1 histology. However, a definitive diagnosis of AIP1 can be achieved based on a combination of clinical, radiological, and serological criteria that do not require histology. Conversely, a definitive diagnosis of AIP2 necessitates histological confirmation due to the absence of specific findings and surrogate markers.

It is important to note that most studies on the role of EUS-guided sampling focus on AIP1, with data on AIP2 being anecdotal and based on small samples [115]. This may be due to the rarity of the disease, particularly in Western countries, which have contributed more to the understanding of this issue. While EUS-FNA was the initial approach to pancreatic biopsy sampling, its results in diagnosing AIP have been less successful compared to PC diagnosis [116,117,118]. In a recent review regarding the role of EUS-guided sampling in patients with AIP, the outcomes of EUS-FNA in diagnosing AIP are disappointing, with level 1 histology achieved between 0% and 56% [115].

Based on the available literature, authors suggest using EUS-FNA to exclude PC rather than to diagnose AIP [115,119,120]. Steroid therapy response is considered a key criterion by the ICDC, especially in patients lacking typical radiological and serological criteria. Excluding PC with negative FNA for malignancy is a prerequisite for steroid administration, which may be necessary for diagnosing AIP [20,24]. If a patient does not respond to a diagnostic corticosteroid trial, definitive diagnosis should be pursued through surgical exploration or resection [66].

As cytological/biopsy negativity does not guarantee non-malignancy, a short-term follow-up (2 weeks) with imaging to evaluate corticosteroid response is necessary [20]. It should be emphasized that repeating EUS-FNA is justified when doubts about pancreatobiliary malignancy persist after non-diagnostic tissue sampling [121]. Furthermore, it is important to consider that AIP is much rarer than PC and CCA [122].

Since the 2000s, new biopsy needles have been introduced, allowing for not only cytological but also histological evaluation. These needles can assess unique histological and immunohistochemical characteristics leading to the histological confirmation of AIP [123,124,125]. The first-generation FNB needle was the reverse side-beveled needle, but disappointing results were reported, with no substantial advantage over standard FNA needles [126]. Recently, newly designed FNB forward-acquiring needles have shown significantly better results than standard EUS-FNA [127,128] and first-generation EUS-FNB needles [129]. These needles include Franseen-tip needles, side-beveled forward-cutting beveled needles (20-gauge caliber available only), fork-tip needles, and Menghini-type needles [115]. Therefore, EUS-guided sampling has shifted from FNA to FNB [130].

In a prospective multicenter study by Ishikawa et al. [131], 56 patients with suspected AIP underwent EUS-guided FNB using a 22-gauge Franseen needle with an average of 2 passes. The authors reported a detection rate of 58.2% for ICDC level 1 histology and 93% for a level 1 or 2 histology of AIP1. Notably, 8 of 13 patients without a definitive preoperative diagnosis of AIP achieved a definitive diagnosis after FNB, gaining a real clinical advantage for 8 of 56 (14%) patients. Interestingly, when the length of the core tissue was 20 mm or more, the level 1 histology rate rose to 85.7%, suggesting that longer core tissue samples may impact the detection rate of histological criteria for AIP [132].

In a recent randomized prospective study by Kurita et al. [133], comparing the first pass of the Franseen needle to the first pass of the 20G forward-bevel needle, the presence of level 1 histologic criteria according to the ICDC was diagnosed in 56% and 26%, respectively (*p* = 0.001).

These results are similar to those of Ishikawa et al. [131] for the detection rate of level 1 histology (56% vs. 58.2%) but not as good for the lymphoplasmacytic infiltration rate (84% vs. 100%) and level 2 histology (22% vs. 34.5%), resulting in a lower rate of level 1 or 2 histology (78% vs. 92.7%).

These two studies have several strengths, including large sample sizes and prospective designs. Both advocate for using FNB in patients with suspected AIP, particularly suggesting the use of a 22-gauge Franseen needle. However, there are limitations. Firstly, both studies included highly selected populations (pre-EUS suspicion of AIP1), so challenging cases with atypical imaging appearance, mass-forming lesions, and potential misdiagnoses with PC were not included. In addition, the clinical advantage of AIP patients with a definitive diagnosis before EUS-guided FNB is debatable [134].

Finally, as mentioned in the aforementioned review [115], all available studies on the ICDC histological criteria for diagnosing AIP are based on surgical specimens and tru-cut biopsies, which are infrequently performed. Therefore, diagnostic criteria should be adapted to tissue fragments obtainable through EUS-FNB, which is currently the standard of care for preoperative pancreatic tissue sampling.

In summary, the superiority of FNB versus FNA in patients with AIP has been reported in three recent systematic reviews [33,135,136], but despite that, the diagnostic accuracy is not excellent. The limitations of pancreatic biopsy sampling in AIP confirm that the diagnosis of AIP should be carefully based on a combination of several criteria established by international guidelines.

## 6. Conclusions

Our work presents a comprehensive literature review on the role of EUS and ancillary techniques in assessing autoimmune pancreatitis. Compared to previous reviews [137,138], we included the latest studies to provide an extensive overview of all EUS techniques, ranging from conventional imaging to pancreatic biopsy, including time–intensity curve analysis and elastography. Although the standard EUS characteristics of AIP are well defined, they are not yet included in diagnostic guidelines. Further studies are needed to clarify the roles of elastography, ultrasonographic contrast, and the time–intensity curve concept.

In summary, the diagnosis of AIP includes the following:

- Requires the exclusion of PC;
- Strongly relies on radiological and EUS imaging. The typical EUS appearance of autoimmune pancreatitis is diffuse pancreatic enlargement, the so-called “sausage-shaped pancreas,” with a hypoechoic, patchy, and heterogeneous echotexture. In the focal form, which accounts for 28–41% of AIP cases, EUS reveals a focal hypoechoic enlargement or area. Other relevant features include typical parenchymal and chronic pancreatitis changes, as well as biliary and peripancreatic findings, all detailed in Table 1. After the injection of Sonovue™, the more typical contrast-enhanced pattern is hyper- or isovascular homogeneous in the arterial phase with a tendency for persistent enhancement in the late phase. The role of TICs is promising, as is elastography, also in assessing the response to therapy, but they are still under investigation.
- EUS-FNB is a valuable tool for diagnosis, but its results remain unsatisfactory, as grade 1 histology cannot be achieved in up to 40% of patients [136]. In such cases, AIP1 diagnosis can be made by combining clinical, radiological, and serological criteria in accordance with the ICDC guidelines and ultimately by the patient’s response to steroids.
- Vascular involvement in AIP appears to be underestimated by EUS, as there is a significant difference in detection rates between EUS and cross-sectional imaging. This discrepancy could serve as a starting point for new studies.

## Figures and Tables

**Table 1:** Endoscopic ultrasonography features of autoimmune pancreatitis.

Pancreas		Extrahepatic Bile Duct	Gallbladder	Lymph Nodes	Peripancreatic Vessels
d-AIP	f-AIP				
**Gland volume:** diffuse enlargement	**Gland volume:** focal enlargement (within the pancreatic head in about 2/3 of cases) [37]	**Caliber:** dilated.	**Wall:** thickened.	**Volume:** enlarged (≥8 mm) [48]	The loss of interface between the pancreas and vessels of the portal system and potentially others [48]
**Echotexture:** diffuse hypoechogenicity, diffuse hypoechoic areas	**Echotexture:** focal or diffuse hypoechogenicity [48]	**Wall:** regular homogenous thickening typically with a hyper–hypo–hyperechoic series of layers (“sandwich pattern”) or parenchymal echo type [62]		**Echotexture:** hypoechoic	
Parenchymal heterogeneity: hyperechoic foci, lobularity ^†,∆^, hyperechoic strands ^∆^					
**MPD:** hyperechoic margin [1], **irregular narrowing**	**MPD:** hyperechoic margin [1], irregular narrowing, upstream dilation [37,48]				
**Peripancreatic changes:** peripancreatic hypoechoic margin [37], lobular outer margin ^∆^	**Peripancreatic changes:** lobular outer margin ^∆^ [37,47]				

d-AIP: diffuse-type autoimmune pancreatitis; f-AIP: focal-type autoimmune pancreatitis; MPD: main pancreatic duct. ^†^ Significantly higher frequency in early-stage disease [51]. ^∆^ Features that improve with steroid therapy [47].

**Table 2:** Characteristic EUS findings of autoimmune pancreatitis in comparison with pancreatic cancer.

	AIP	Pancreatic Carcinoma
**Conventional EUS**	Diffuse hypoechogenicity Diffuse or focal hypoechoic areas Hyperechoic foci/strands Lobularity Peripancreatic hypoechoic margin Duct-penetrating sign **^†^** Hyperechoic MPD margin Irregular MPD narrowing **^∆^** Bile duct wall thickening ° Lymphadenopathy ^	Focal hypoechogenicity MPD dilation Vascular invasion
**Contrast-enhanced EUS**	Hyper-isoenhancement Homogeneous contrast agent distribution Absent irregular internal vessels [80]	Iso-hypoenhancement Arterial irregularity and absent venous vasculature [80]
**Elastography**	Homogeneous pattern (small spotted mainly blue) spread over the pancreatic parenchyma, not just at the mass [38]	Focal heterogeneous pattern (predominantly blue with small green areas and red lines and a geographic appearance) [113]

AIP: autoimmune pancreatitis; EUS: endoscopic ultrasound; MPD: main pancreatic duct. ^†^ High specificity but insufficient sensitivity for focal-type AIP [48,59]. ^∆^ Association with main pancreatic duct wall thickening has shown overall accuracy of 98.3% for AIP [59]. ° Intrahepatic and/or hilar bile duct strictures are important clues for distinguishing AIP from pancreatic carcinoma [66]. ^ Trend toward higher prevalence of lymphadenopathy in AIP compared to pancreatic carcinoma was observed [48].

**Table 3:** Histological features and diagnosis of AIP according to International Consensus Diagnostic Criteria.

	Level 1	Level 2
**AIP1**	LPSP At least 3 of the following: (1) Periductal lymphoplasmacytic infiltrate without granulocytic infiltration (2) Obliterative phlebitis (3) Storiform fibrosis (4) IgG4-positive cells > 10/HPF	LPSP Any 2 of the following: (1) Periductal lymphoplasmacytic infiltrate without granulocytic infiltration (2) Obliterative phlebitis (3) Storiform fibrosis (4) IgG4-positive cells > 10/HPF
**AIP2**	IDCP Both of the following: (1) Granulocytic infiltration of duct wall with or without granulocytic acinar inflammation (2) Absent or scant (0–10 cells/HPF) IgG4-positive cells	Both of the following: (1) Granulocytic and lymphoplasmacytic acinar infiltration (2) Absent or scant (0–10 cells/HPF) IgG4-positive cells

AIP: autoimmune pancreatitis; LPSP: lymphoplasmacytic sclerosing pancreatitis; IDCP: idiopathic duct-centric pancreatitis; HPF: High-power field.
